# Variable effects of maternal and paternal–fetal contribution to the risk for preeclampsia combining GSTP1, eNOS, and LPL gene polymorphisms

**DOI:** 10.3109/14767058.2010.511351

**Published:** 2010-09-14

**Authors:** Kalliopi I. Pappa, Maria Roubelakis, George Vlachos, Spyros Marinopoulos, Antonia Zissou, Nicholas P. Anagnou, Aris Antsaklis

**Affiliations:** 1 First Department of Obstetrics and Gynecology, University of Athens School of Medicine, Alexandra University Hospital, Athens, Greece; 2 Biomedical Research Foundation of the Academy of Athens (IIBEAA) and Laboratory of Biology, University of Athens School of Medicine, Athens, Greece

**Keywords:** Preeclampsia genetics, GSTP1 gene, eNOS gene, LPL gene, paternal–fetal contribution, transmission disequilibrium test

## Abstract

**Objective:**

To evaluate the maternal, paternal, and fetal genotype contribution to preeclampsia.

**Study design, materials, and methods:**

We combined the analysis of polymorphisms of the GSTP1, eNOS, and LPL genes – affecting biotransformation enzymes and endothelial function – in a cohort of 167 preeclamptic and normal control trios (mother, father, and child) comprising a total of 501 samples in the Greek population, never analyzed before by this approach.

**Results:**

For the frequency of the GSTP1 Ile^105^/Val^105^, the eNOS Glu298Asp and the LPL-93 polymorphisms, statistically significant differences were found between the two groups. However, the transmission rates of the parental alleles to neonates studied by the transmission disequilibrium test, disclosed no increased rate of transmission to preeclampsia children for the variant alleles of Val^105^ GSTP1, 298Asp eNOS, and -93G LPL.

**Conclusions:**

These novel data, suggest that interaction of all three types of genotypes (mother, father and neonate), reveals no effects on the development of preeclampsia, but provide the impetus for further studies to decipher the individual contribution of each genetic parameter of preeclampsia.

## Introduction

Preeclampsia represents the clinical expression of a complex metabolic disorder during pregnancy, affecting 3–10% of all pregnancies [1]. It is characterized by high maternal and fetal morbidity and therefore, constitutes a high-risk condition of pregnancy [2]. The main cause of this major complication is still unknown. It is believed that genetic, epigenetic, and environmental parameters are involved in the pathogenesis of preeclampsia [3,4]. The most interesting theory refers to the concept of an imbalanced lipid peroxidation state, leading to the generation of reactive oxygen species and toxic substrate accumulation, resulting eventually in preeclampsia [2,5–9].

Glutathione S-transferase P1 (GSTP1), endothelial nitric oxide synthase (eNOS), and lipoprotein lipase (LPL) constitute three major detoxification enzymes that have been studied in pregnancy, as putative predisposing factors to preeclampsia [10,11].

Glutathione S-transferase P1 transfers aliphatic, heterocyclic radicals, epoxides, aren oxides substrates, and xenobiotics to glutathione [12]. Glutathione S-transferase P1-1 isoform (GSTP1-1) represents the main isoform in placenta and decidua and exhibits lower levels in preeclamptic pregnancies [13]. So far, two variant cDNAs have been reported differing at one base pair (313 A-to-G transition), resulting in an isoleucine to valine substitution at position 105 (105 Ile→Val) and thus in a less functional enzyme [14,15]. Therefore, Val 105 polymorphism results in lower detoxification in the trophoblast and thus increases the oxidative status and susceptibility to preeclampsia [16].

Nitric oxide (NO) and its signaling cascade are important for the placental function and particularly for the regulation of blood flow and vasomotor tone [17]. It is synthesized by the endothelial nitric oxide synthase (eNOS). The eNOS Glu298Asp gene polymorphism has been reported to be associated with severe preeclampsia [18,19], but not in all populations studied [20–22]. Polymorphisms of the eNOS gene detected in 9–13% of the general population may affect its functional capacity. The common missense variant Glu298Asp polymorphism seems to enhance enzyme proteolytic capacity and results to low NO bioactivity [23] and reduced endothelium-dependent vasodilation.

LPL hydrolyzes and releases the free fatty acids from the very low-density lipoproteins (VLDL) and chylomicrons [24]. LPL deficiency results in aberrant fatty acid metabolism. Many mutations have been described so far and their frequency has been estimated to be 1:500 individuals [24–26]. Furthermore, mutations in the coding region of the LPL gene have been associated with dyslipidemia [27,28]. The most frequent mutation is the substitution of T to G at the proximal promoter region, i.e. (-93 position), resulting in a functional variant with aberrant transcriptional activity, associated with coronary artery disease, dyslipidemia, atherosclerosis, and preeclampsia [28,29]. However, this variant has not been associated with an increased risk for preeclampsia in white North American patients [30].

A large Swedish cohort population study showed that maternal genes may contribute to preeclampsia by 35%, fetal genes by 20%, while 13% was attributable to the couple effect, 32% to undetermined factors and less than 1% to shared sibling environment [31]. The fetus contribution is affected by paternal genes; on the contrary, maternally transmitted mitochondrial genes do not contribute to the risk [32]. Extensive genotyping studies [33,34] excluded several genetic variants as putative risk factors, while they documented that a paternal, but not maternal history of essential hypertension is associated with increased risk of nonpregnant hypertension in the children. Thus, placenta and the fetus reflect in part, the paternal component of the pregnancy and their unique genotypes may contribute to the development of preeclampsia, under a specific maternal genotype. However, this rather new approach of evaluation of the role of paternal–fetal genotype on the susceptibility for preeclampsia has led to inconsistent and conflicting results in the few studies conducted so far [16,33–37]. The reasons for this failure might reflect analysis of individual single-gene polymorphisms, or evaluation of genes not linked pathophysiologically to the mechanisms of preeclampsia, or the effects of different genetic backgrounds of the ethnic groups analyzed, or finally, small size of samples. Therefore, it is necessary to evaluate and delineate more systematically the contribution of the maternal and fetal genotypes, by using a combined analysis of a series of candidate genes directly involved in the pathophysiology of preeclampsia.

To this end, and based on previous data, we designed a case–control study to evaluate the contribution of maternal and paternal–fetal components for the risk of preeclampsia, by analyzing the effects on preeclampsia risk of the three relatively common polymorphisms of the GSTP1, eNOS, and LPL genes in a cohort of preeclamptic and normal control trios (mother, father, and child) belonging to a different ethnic population (i.e. Greek) never analyzed previously by this approach. We hypothesized that these gene polymorphisms might constitute a basis for the triggering events leading to preeclampsia, by affecting the activity of biotransformation enzymes (GSTP1 and eNOS) in detoxifying processes and/or the endothelial function (LPL).

## Materials and methods

### Patients and study subjects for DNA polymorphisms

The prospective case–control study enrolled 167 unrelated pregnant women participating as trios (mother, father, and neonate), 51 (30.95%) with preeclampsia, and 116 (69.05%) healthy pregnant women, as controls. Thus, the total population studied comprised of 501 samples. Subjects were recruited from the Obstetric Clinic of the First Department of Obstetrics and Gynecology, at the University of Athens School Medicine. All pregnant women were on singleton pregnancy, while the fetuses of polypara preeclamptic women were fathered by the same father. Participants' mean age was 27.6 + 4.7 years. None of the women were on medication, except of a subgroup of cases, requiring regimen for maintaining normotensive status. Each participating individual provided informed consent. Institutional Review Board approval for the study was obtained by the University Hospital Ethics Committee.

Basic demographic and clinical parameters, such as gestational age, height, body weight and body mass index (BMI) before pregnancy, systolic and diastolic blood pressure, liver enzymes, urine protein, serum creatinine, platelet count, right upper quadratic pain, edema, and visual disorders, were recorded for all subjects. All pregnant women underwent serum and urine specimen analysis before labor.

Preeclampsia was defined as the occurrence, after the 20th gestational week, of systolic blood pressure greater than 140 mm Hg and of diastolic blood pressure greater than 90 mm Hg on two or more recording times with at least 4 h apart, combined with proteinuria with urine protein excretion greater than ≥0.3 g/day or ≥2 + on one voided dipstick testing of midstream urine. Women with hypertension prior to pregnancy, diabetes mellitus, renal or cardiac disease, were excluded from the study.

On the basis of the aforementioned criteria, the definitive diagnosis for preeclampsia was made at or after the 26th gestational week, while following delivery, 5 ml of venous blood was drawn from each one of the triplet (mother, father, and neonate) and collected in EDTA-containing tubes. The samples were stored at −80°C until analyzed. Every triplet was studied for eNOS, GSTP1, and LPL polymorphisms.

### DNA extraction and analysis of gene polymorphisms

Genomic DNA was extracted from whole blood using the NucleoSpin Blood Kit (Macherey-Nagel, Bioanalysis, GmbH & Co. KG, Düren, Germany), according to the instructions of the manufacturer. The procedure typically resulted in the isolation of 50–200 ng of genomic DNA.

**Figure 1:**
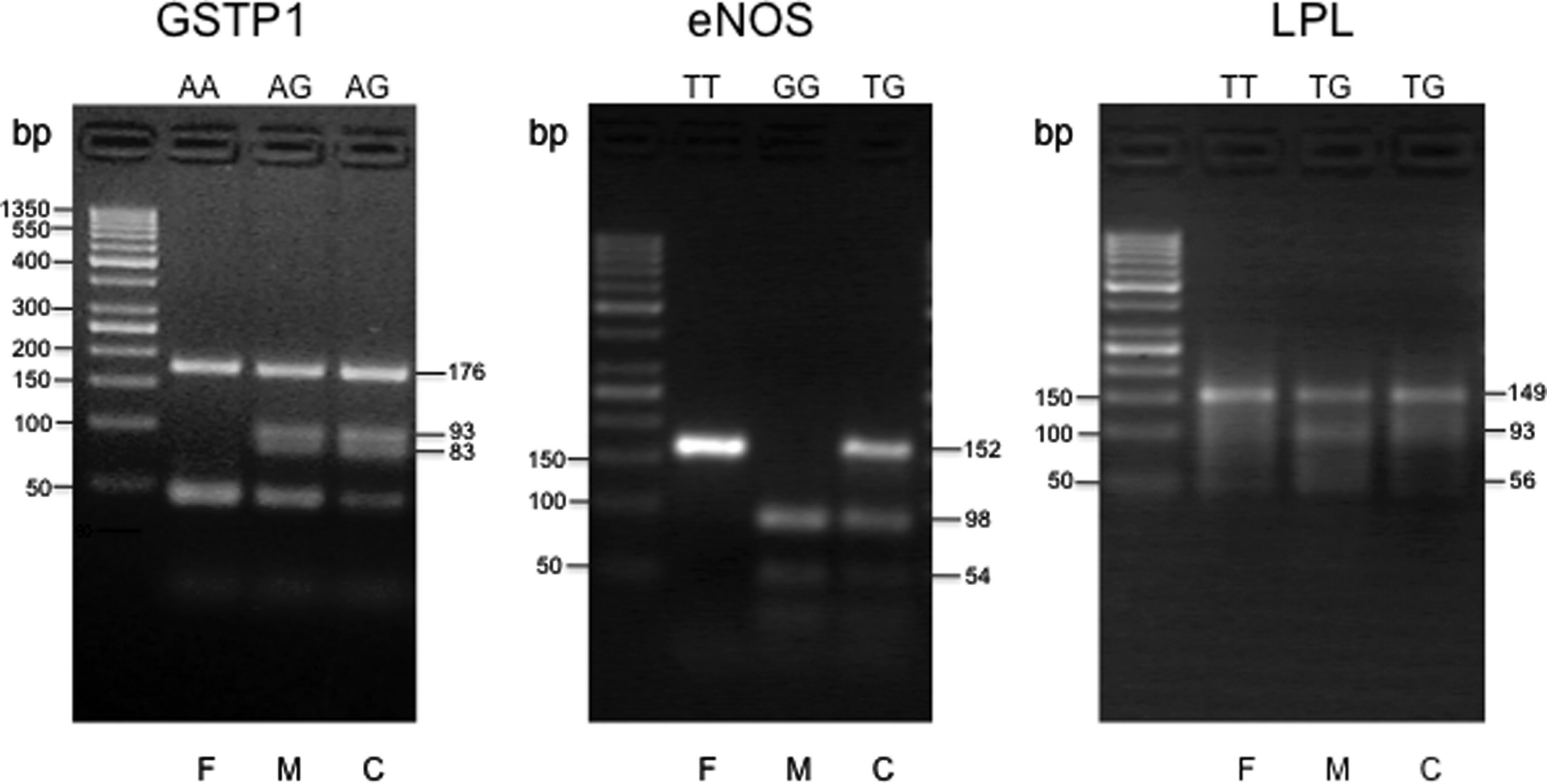
Evaluation of the polymorphisms for the GSTP1, eNOS, and LPL genes, using a combination of PCR analysis, followed by restriction enzyme digestion and resolution of the digested PCR products by electrophoresis in 4% agarose gels. Representative trios (father, F; mother, M; and child, C) with their respective genotypes for each gene are shown. The size of the expected digested products, in base pairs (bp), is shown on the right of each panel. The first lane of each panel contains bands of the 50 bp DNA ladder, ranging from 50 to 1350 bp, used as molecular weight standards.

Polymorphisms for glutathione S-transferase P1 (GSTP1), endothelial nitric oxide synthase (eNOS), and lipoprotein lipase (LPL) genes were detected by a combination of polymerase chain reaction (PCR) and restriction fragment length polymorphism (RFLP) assays, using appropriate restriction enzymes as described previously [16,23,29]. The primers used (forward and reverse) for the polymorphic loci are shown in Table I. All chemicals for PCR were purchased from Minotech (FoRTH, Heraklion, Greece). For the evaluation of polymorphisms, amplified products were resolved on a 4% agarose gel electrophoresis, as shown in Figure 1.

**Table I:** Primer pair sequences used in PCR for the detection of the three gene polymorphisms.

Gene	Marker	Location Primer (5′ to 3′)
GSTP1	Exon 5	Forward 5′-ACCCCAGGGCTC TATGGGAA-3′
		Reverse 5′-TGAGGGCACAAGA AGCCCCT-3′
eNOS	Exon 7	Forward 5′-AGGAAACGGTCG CTTCGACGTGCTG-3′
		Reverse 5′-CCCCTCCATCCCA CCCAGTCAAC-3′
LPL	-93 position of promoter region	Forward 5′-GGCAGGGTTGAT CCTCATTACTGTT-3′
		Reverse 5′-GACACTGTTTTCA CGCCAAGGCTGC-3′

The PCR reaction was carried out in a total volume of 30 µl in a PTC-200 Peltier Thermal Cycler (MJ Research, Waltham, MA). The PCR reaction mixture contained 3 µl 10 × reaction buffer, 1 µl dNTP mix (10 mM of each dNTP), 1 µl forward primer (25 pmol), 1 µl reverse primer (25 pmol), 1.8 µl MgCl_2_ (25 mM), 1 µl Taq DNA polymerase (5 u/µl), and 5 ng/µl DNA template. Amplification was carried out with 5 min initial denaturation at 95°C, followed by 35 cycles of denaturation at 95°C for 30 s, primer annealing at the appropriate temperature for each primer for 30 s, and extension at 72°C for 1 min.

For GSTP1, the PCR yielded a 176-bp product, and following digestion with the restriction enzyme BsmA I, an isoschizomer of Alw26 I (New England BioLabs, Beverly, MA), yielded a 93-and a 83-bp product for the G/G genotype, a 176-, a 93-, and a 83-bp product for the A/G genotype, while the enzyme did not cut the normal A/A genotype sequence (Figure 1).

For eNOS, the PCR yielded a 152-bp product, which, after digestion with the restriction enzyme Ban II (New England BioLabs), yielded a 98-and a 54-bp fragment for the normal G/G genotype, a 152-, a 98-and a 54-bp fragment for the T/G genotype, while the PCR product with the T/T genotype was not digested (Figure 1).

For the LPL-93 polymorphism, the PCR yielded a 149bp product, which following digestion with the restriction enzyme Ban II, yielded a 93-and a 56-bp fragment for the G/G genotype, a 149-, a 93-, and a 56-bp fragment for the T/G genotype, while the PCR product with the normal T/T genotype was not digested (Figure 1).

### Statistical analysis

For the statistical analysis, the nonparametric tests of two-sample Wilcoxon matched-pairs rank-sum test and the χ^2^ Fisher's exact test were applied accordingly. For the statistical analysis of the polymorphisms, the χ^2^ Fisher's exact test was used. The number of parental–fetal allele transmissions compared to the number of transmissions expected by chance was studied using the transmission disequilibrium test (TDT) [38]. The level of statistical significance was defined as *P* < 0.05.

## Results

### Clinical and biochemical features of the two groups of pregnant women

The initial total population studied comprised of 504 individuals (i.e. 168 mothers, their corresponding 168 husbands, and 168 children) from each trio. Because of the lack of information for one child, 167 trios were finally evaluated for genotyping.

The main demographic and clinical features of the two groups of pregnant women (i.e. preeclamptic and normal controls) are shown in Table II. All the clinical and biochemical parameters between the two groups with the exception of platelet count were statistically significant. Specifically, the prepregnancy BMI, systolic and diastolic blood pressure, serum GOT, GPT, and creatinine levels were higher in the preeclamptic group, and all differences were statistically significant (*P* < 0.001), as shown in Table II. Furthermore, all the additional quantitative clinical parameters studied, such as right-upper quadrant pain (P = 0.009), edema (*P* < 0.001), headache (*P* < 0.001), visual disorders (*P* < 0.001), and urine protein (*P* < 0.001), were found to be statistically significant between the two groups, using the χ^2^ Fisher's exact test.

**Table II:** Selected demographic and clinical features of the two groups of pregnant women.

	Preeclampsia (*n* = 51)	Normal conthols (*n* = 116)	*P*-value*
Age (years)**	26 (18–36)	27 (21–37)	0.023
Pre-pregnancy BMI (kg/m^2^)**	25.2 (18.6–34.7)	21.5 (18.4–31.1)	<0.001
Systolic blood pressure (mm Hg)**	159.0 (100–230)	110 (100–120)	<0.001
Diastolic blood pressure (mm Hg)**	100 (70–150)	75 (65–80)	<0.001
Serum GOT† (U/l)**	23 (7–129)	13 (9–34)	<0.001
Serum GPT‡ (U/l)**	20.0 (7–466)	12 (6–52)	<0.001
Creatinine (mg/dl)**	0.70 (0.36–1.30)	0.60 (0.40–0.70)	<0.001

* The comparison between the two groups, due to the non-normal distribution of the variables, was performed using the nonparametric criteria of the two-sample Wilcoxon matched-pairs rank-sums test, which is equivalent to the Mann–Whitney test.

** The appropriate values of median and range were used, since the distribution of these continuous variables is not normal.

† GOT, glutamic oxaloacetic transaminase.

‡ GPT, glutamic pyruvic transaminase.

### Genotypic frequencies of the three polymorphic alleles in the two groups

We next studied the distribution of the frequencies of GSTP1, eNOS, and LPL genotypes in the subgroups of the mothers, fathers, and children of the preeclampsia and normal control groups. The comparisons were performed using the Fisher's exact test. The distribution of the polymorphic variants of the three genes in the preeclampsia trios and the normal control group is shown in Table III.

**Table III:** Distribution of the GSTP1, eNOS, and LPL polymorphisms in mother, father, and neonate trios of the two groups.

		*n* (%)		*P*-value*
		
	Genotype	Preeclampsia	Normal controls	
GSTP1 *gene*				
Mother	Ile^105^/Ile^105^	34 (66.67)	68 (58.62)	
	Ile^105^/Val^105^	15 (29.41)	36 (31.03)	0.013
	Val^105^/Val^105^	2 (3.92)	12 (10.34)	
Father	Ile^105^/Ile^105^	28 (54.90)	68 (58.62)	
	Ile^105^/Val^105^	23 (45.10)	38 (32.76)	0.001
	Val^105^/Val^105^	0 (0.00)	10 (8.62)	
Child	Ile^105^/Ile^105^	38 (74.51)	69 (59.48)	
	Ile^105^/Val^105^	11 (21.57)	38 (32.76)	0.007
	Val^105^/Val^105^	2 (3.92)	9 (7.76)	
eNOS *gene*				
Mother	298Glu/298Glu	22 (43.14)	57 (49.14)	
	298Glu/298Asp	29 (56.86)	49 (42.24)	0.001
	298Asp/298Asp	0 (0.00)	10 (8.62)	
Father	298Glu/298Glu	27 (52.94)	44 (37.93)	
	298Glu/298Asp	24 (47.06)	64 (57.17)	0.002
	298Asp/298Asp	0 (0.00)	8 (6.90)	
Child	298Glu/298Glu	24 (47.06)	50 (43.10)	
	298Glu/298Asp	25 (49.02)	57 (49.14)	0.025
	298Asp/298Asp	2 (3.92)	9 (7.76)	
LPL *gene*				
Mother	−93T/−93T	46 (90.20)	113 (97.41)	
	−93T/793G	5 (9.80)	3 (2.59)	0.047
Father	−93T/−93T	49 (96.08)	113 (97.41)	
	−93T/793G	2 (3.92)	3 (2.59)	0.317
Child	−93T/−93T	49 (96.08)	111 (95.69)	
	−93T/793G	1 (1.96)	4 (3.45)	0.153
	793G/793G	1 (1.96)	1 (0.86)	

* *P*-values were generated using χ^2^ Pearson's test. Where necessary, the corresponding Fisher's exact test was also used.

For the GSTP1 polymorphism, the frequency of the variant Val^105^ allele, although previously reported to be associated with preeclampsia [16] was found to be higher in mothers (25.8%) and fathers (25.0%) of normal pregnancies compared to the preeclamptic group, while a statistically significant difference was detected between the frequency of the individual subgroups compared to that of the normal controls (*P* = 0.013 and *P* = 0.001, respectively). It is noteworthy, that no homozygote for the Val^105^ allele was found among the fathers of the preeclampsia trios (Table III).

For the eNOS Glu298Asp polymorphism, a similar pattern of frequency distribution was observed (Table III). The frequency of the 298Asp allele was higher in mothers (29.7%), fathers (34.4%) and children (32.3%) of the normal control group compared to the preeclamptic group and a statistically significant difference was detected between the individual subgroups (*P* = 0.001, *P* = 0.002, and *P* = 0.025, respectively). No homozygote individual for the 298Asp/298Asp genotype was found in the group of preeclamptic mothers and fathers.

For the -93LPL polymorphism of the promoter region, the frequency of the variant -93G allele was higher in mothers (4.9%), fathers (1.9%), and children (2.9%) of preeclamptic pregnancies compared to the normal control subgroups. However, when the frequencies of the individual subgroups of preeclampsia were compared to those of the normal controls, a statistically significant difference was documented only for the mothers (*P* = 0.047), while no statistically significant difference was documented between the subgroups of fathers (*P* = 0.317) or of the children (*P* = 0.153).

### Transmission pattern of the preeclamptic parental allele to neonates

We next investigated the mode of transmission of the associated marker allele from a heterozygous parent to an affected, in this case, neonate of a preeclamptic pregnancy. To this end, we have utilized the transmission disequilibrium test or TDT [38] developed as a test for linkage between a complex disease, such as preeclampsia and a genetic marker, such as the polymorphic alleles of the GSTP1, eNOS, and LPL genes. The TDT actually evaluates the observed number of parent–child transmissions of alleles, compared to the number of transmissions anticipated by chance. The TDT test was applied in cases where at least one parent was heterozygote for the polymorphism, since only heterozygotes are informative for the test.

For the GSTP1 polymorphism, a total of 56 transmissions of the variant Val^105^ allele, and 118 transmissions of the common lle^105^ allele, from 87 parents were observed. The application of the TDT test was found to be not statistically significant (*P* = 0.074), indicating that there is no difference in the rate of transmission of the common lle^105^ allele versus the variant allele Val^105^ to preeclampsia children.

For the eNOS polymorphism, 87 transmissions of the variant 298Asp allele and 151 transmissions of the common 298Glu allele were observed in a total of 119 parents. The TDT test was not statistically significant (*P* = 0.677). Therefore, there is no increased transmission of the variant 298Asp allele to the preeclampsia children.

For the -93LPL polymorphism, six transmissions of the variant -93G allele and 16 transmissions of the common -93T allele were observed in 11 parents. The TDT test was not found to be statistically significant (*P* = 1.00). Therefore, the data suggest no increased transmission frequency of the variant allele to preeclampsia children.

## Discussion

Maternal susceptibility to preeclampsia is a multifactorial and complex process, involving the contribution of a series of essential gene variants [3,4,39], affecting hemodynamic and vascular parameters, oxidative stress pathways causing endothelial damage and induction of metabolic and coagulation aberrations.

Furthermore, the central role of placenta and the epigenetics of the placentally expressed genes are currently recognized as the cardinal features of the pathophysiology of preeclampsia [3,4]. Specifically, expression analysis of preeclamptic placental genes has revealed a group of numerous target genes putatively involved in abnormal placentation [40] and another set of 13 genes significantly upregulated, with functions involving tumor suppression, growth regulation, and inhibition of trophoblast invasion [41]. These gene sets can be further evaluated for their triggering role in preeclampsia.

Although such studies are expected to delineate the multivariable parameters of preeclampsia mechanisms, additional complementary approaches are also needed to address the issues of the individual contribution of the parental and fetal genotypes to the risk for preeclampsia. Familial studies have convincingly supported the hypothesis that transmission of the father's genes to the fetus (actually to placenta) can indeed increase the risk for preeclampsia [32,42]. More recent studies using linked birth data among family members in Norway [36] and in Malay women in Singapore [37], have further provided evidence that both maternal and fetal genes either from the mother or the father, can trigger the mechanisms of preeclampsia. Thus, maternal susceptibility seems to be mediated by a combination of maternal susceptibility genes and by an independent transmission to the fetus of genetic risk factors. It is of interest, that in the Norwegian study [36] the data strongly suggested that there was a differential contribution between the maternal and paternal components; mothers seem to carry both susceptibility genes and also transmit independent genetic risk factors to the fetus, while fathers transmit only fetal risk factors. The validity of these findings, have been followed up by a series of large-scale multicenter candidate gene studies [33,34] aiming to identify fetal and maternal susceptibility for preeclampsia, involving extensive genotyping of seven candidate genes, previously considered to confer susceptibility to preeclampsia. However, none of the seven selective genetic maternal and fetal variants were found to confer a high risk for preeclampsia. This failure, probably reflects, among other reasons, the lack of inclusion of additional preeclampsia-specific genes [10,43] which could eventually provide a better understanding of the pathogenetic mechanisms of preeclampsia.

It is therefore evident that a comprehensive approach is needed to decipher the mechanisms of susceptibility to preeclampsia, by encompassing all the genetic components involved in the formation of placenta (i.e. maternal, paternal, and fetal genotypes). This new approach has been used so far in a single study to address the effect of the paternal contribution, by studying the GSTP1 gene polymorphism in a cohort of 113 preeclampsia trios in the Dutch population [16]. The paternal and fetal polymorphism of the GSTP1 gene was found to be associated with a high risk for preeclampsia [16]. This polymorphism had been associated earlier by the same group [14] in the maternal genome of preeclamptic women, while no association had been found in either preeclampsia, eclampsia or HELLP syndrome in the maternal genome of a different ethnic group for a polymorphism of two closely related cytosolic soluble glutathione S-transferase genes of the mu (M) and theta (T) families [44]. It is of interest that homozygosity for the Val^105^ polymorphism was associated with severe preeclampsia because of the production of a GSTP1 enzyme with reduced detoxifying capacity [15,16,45,46]. In contrast, another study [47] has reported a high frequency of the Val^105^/Val^105^ genotype in maternal and fetal samples in a South African population, but with no proportional increase in preeclampsia. It has also been speculated that the GSTP1 variant contributes to preeclampsia phenotype despite the fact that the frequency of the Ile^105^/Val^105^ and Val^105^/Val^105^ genotypes was not significantly different between the preeclampsia and the control group [48].

On the basis of these limited and conflicting studies, we designed this study to evaluate more systematically the contribution of maternal, paternal, and fetal components for the risk of preeclampsia, by selecting three gene polymorphisms, affecting the activity of biotransformation enzymes, such as GSTP1 and eNOS, and the endothelial function, such as LPL, in an ethnic population (i.e. Greek) never analyzed previously by this comprehensive approach. We hypothesized that detoxification and endothelial function-linked paternal gene variants inherited by the fetus are expressed in the fetal–placental unit, and under a specific background of maternal genotype, they might trigger a hyperoxidative status, which is eventually manifested as preeclampsia.

The genotypic distribution and the frequencies of the three polymorphic alleles between the preeclampsia and the normal control trios revealed several informative features between the two groups.

For the GSTP1 polymorphism, the frequency of the variant Val^105^ allele interestingly was found to be higher in mothers and fathers of normal pregnancies compared to the preeclampsia group, a finding nonconsistent with previous data [16]. Furthermore, this difference was statistically significant, and the TDT test applied to the heterozygote parents for the variant allele Val^105^, disclosed no difference in the rate of transmission versus the common Ile^105^ allele to preeclamptic children. These data are in contrast to those performed on the Dutch population [16]. These discrepancies between the two studies apparently reflect different allele frequencies between the two ethnic populations and discrete genotype backgrounds regarding the individual putative alleles contributing to preeclampsia. Actually, candidate gene studies underscore the fact that single-gene effects may be variable across populations [4,49,50].

For the eNOS polymorphism, the frequency of the variant 298Asp allele was found to be statistically significant between the two groups of trios, exhibiting higher frequency in the control group. Furthermore, the TDT test applied to 87 transmission patterns was not statistically significant. These data represent the first attempt to evaluate the contribution of all three types of eNOS genotypes in a preeclamptic group. Previous studies, using analysis of simple maternal genotypes, have associated the 298Asp allele with severe preeclampsia in some [18] but not in all populations studied [20]. It should be noted also that this variant allele is represented in 9–13% of the general population and seems to affect the proteolytic activity and bioactivity of NO [23], resulting in reduced endothelium-dependent vasodilation, a state that can lead to preeclampsia.

In addition, a different polymorphism of the eNOS gene within intron 4 (designated as allele A) has been shown to be associated with preeclampsia in a defined ethnic group [51]. The odds ratio of developing preeclampsia when at least one A allele was present was 6.5, suggesting that this polymorphism might have predictive value for preeclampsia. Finally, a recent extensive meta-analysis of the eNOS Glu298Asp polymorphism involving 1055 patients and 1788 controls did not reveal any clear association with preeclampsia [52]. Therefore, the data from our studies and those of other investigators imply that the eNOS polymorphism per se is not sufficient to clearly confer predisposition to preeclampsia. Further studies are thus needed to clarify additional regulatory parameters of the eNOS-NO activity in normal placenta, such as novel peptides, i.e. adiponectin, apelin, and ghrelin 91 and 92 that utilize the NO pathway to modulate vascular tone [17].

Regarding the frequency distribution and the effect of paternal–fetal transmission of the LPL gene polymorphic genotypes, consistent frequency differences of the two alleles between the subgroups of the two categories were documented, with the frequency of the variant -93G allele being higher in mothers, fathers, and children of preeclamptic pregnancies. These data are consistent with previous findings in Caucasian women concerning the frequency and association of the variant -93G allele with preeclampsia [29]; however, this variant did not exhibit an increased risk in another subsequent study in white North American women [30]. Furthermore, the transmission analysis using the TDT test used for the first time in our study disclosed that there was no increased transmission frequency of the variant allele to preeclamptic neonates.

Since dyslipidemia, a state that predisposes to preeclampsia, reflects a multifactorial and multigene disorder, it is not surprising that the limited data so far on -93LPL polymorphism, based solely on the analysis of maternal genotypes, have led to inconsistent results, suggesting either association to preeclampsia [28,29,53] or no association, when tested in a series of white North American patients [30].

In conclusion, we have used a comprehensive approach for the delineation of the individual maternal, paternal, and fetal genotypes for the risk of preeclampsia, utilizing for the first time the combined analysis of three key gene polymorphisms, affecting the activity of biotransformation enzymes (GSTP1 and eNOS) in detoxifying processes and/ or the endothelial function of placenta (LPL) in a different ethnic population never analyzed before by this approach.

These novel data, based on the transmission test for linkage disequilibrium (TDT), suggest that analysis of the interaction of all three types of genotypes (mother, father, and neonate) from the preeclampsia and control groups reveals variable effects. The GSTP1 polymorphism with its variant Val^105^ allele is not sufficient to clearly confer an increased risk for preeclampsia, neither the eNOS polymorphism with the variant 298Asp allele, nor the LPL gene polymorphism with the -93G variant allele.

Our studies using a different approach, document the molecular and racial heterogeneity of preeclampsia, and provide the impetus for further studies involving (i) the analysis of additional genomic regions revealed by genome scan meta-analysis [4] and (ii) employing large-scale genome-wide association studies [3], to decipher the individual contribution of each genetic and epigenetic parameter [11] of preeclampsia.
